# Clinical Features and Surgical Treatment of Synchronous Multiple Primary Lung Adenocarcinomas With Different EGFR Mutations

**DOI:** 10.3389/fonc.2021.785777

**Published:** 2022-01-13

**Authors:** Rirong Qu, Fan Ye, Dehao Tu, Yixin Cai, Xiangning Fu

**Affiliations:** Department of Thoracic Surgery, Tongji Hospital, Tongji Medical College, Huazhong University of Science and Technology, Wuhan, China

**Keywords:** synchronous multiple primary lung adenocarcinomas, surgical treatment, epidermal growth factor receptor, lung cancer, clinical features

## Abstract

**Background:**

With the popularity of lung cancer screening and advances in imaging technology, more and more synchronous multiple primary lung adenocarcinomas (SMPLA) are being diagnosed clinically, however, the clinical characteristics and prognosis of SMPLA with different EGFR mutations remains unclear. We aimed to explore clinical features and surgical outcomes of these patients to aid in the diagnosis and treatment of SMPLA.

**Methods:**

Medical records of patients with different EGFR mutations who have been diagnosed as SMPLA and underwent surgical resection from March 2015 to December 2019 were retrospectively analyzed. Clinical characteristics, surgical outcomes, recurrence-free survival (RFS) and overall survival (OS) were investigated.

**Results:**

A total of 70 patients (68.6% female and 77.1% non-somkers) were included. Total of 161 lesions in all patients, 84.4% were ground-glass opacity (GGO) lesions. EGFR mutations were detected in 108 lesions, most of which were L858R (35.4%) and 19Del (20.5%). The mutation rate of mixed GGO is significantly higher than that of pure GGO and solid nodules (SN); the mutation rate of invasive adenocarcinoma is significantly higher than that of other histology subtypes; the mutation rate of lesions >20 mm was significantly higher than that of ≤20 mm. However, there is no significant difference in the mutation rate of specific driver gene between different radiological features, pathological characteristics and sizes. After a median follow-up time of 29 months, the 3-year OS and RFS were 94.4% and 86.0%, respectively.

**Conclusions:**

A high discordance of EGFR mutations were identified between tumors in patients with SMPLA. Synchronous multiple lung adenocarcinomas with predominantly multiple GGO should be considered as SMPLA, and surgery may be aggressively performed for these patients due to a good prognosis.

## Introduction

Synchronous multiple primary lung cancer (SMPLC) defined as two or more primary tumors simultaneously identified in ipsilateral or contralateral lung, is a special type of lung cancer. According to previous studies, its incidence varies from 0.2% to 20% (1), of which 40.3%-91.3% (2–4) are multiple primary lung adenocarcinomas. In recent years, the detection rate of SMPLC has shown a steady increase with the popularity of lung cancer screening and advances in imaging technology, especially the widespread use of HRCT and PET-CT (5, 6). Although Surgical resection has become the mainstay of treatment for SMPLC, its 3-year survival rate roughly ranges from 40% to 92% (7). The wide variation in the efficacy of surgical resection is due not only to differences in the timing of treatment, the specific surgical procedures, and demographic characteristics of patients, but more importantly, to the lack of standard criteria for differential diagnosis from intrapulmonary metastasis.

Martini and Melamed’s criteria and ACCP guidelines are most commonly used to distinguish multiple primary lung cancers from intrapulmonary metastases. Because there was no genetic approach to consider in 1975, the Martini-Melamed criteria (8) relied heavily on the clinicopathological features, which can make diagnosis extremely difficult when the tumors are of the similar pathologic type. The ACCP guidelines (9), however, took into account the differences in tumor driver mutation genes, which led to a greater improvement in the diagnosis of multiple primary lung cancers. In addition, the widespread use of next-generation gene sequencing (NGS) in recent years has made the diagnosis of multiple primary lung cancers more accurate (10, 11). More and more SMPLA with different EGFR mutations are being diagnosed clinically, however, the clinical characteristics and prognosis of such patients with surgical treatment remains unknown. Moreover, the reports of such patients are mostly case reports (12–14), and there are few studies with larger samples.

Therefore, in the present study, we focused on the clinical characteristics, surgical outcomes, recurrence-free survival (RFS) and overall survival (OS) of SMPLA with different EGFR mutations to aid in the diagnosis and treatment of these patients.

## Materials and Methods

### Patients

**Figure 1:**
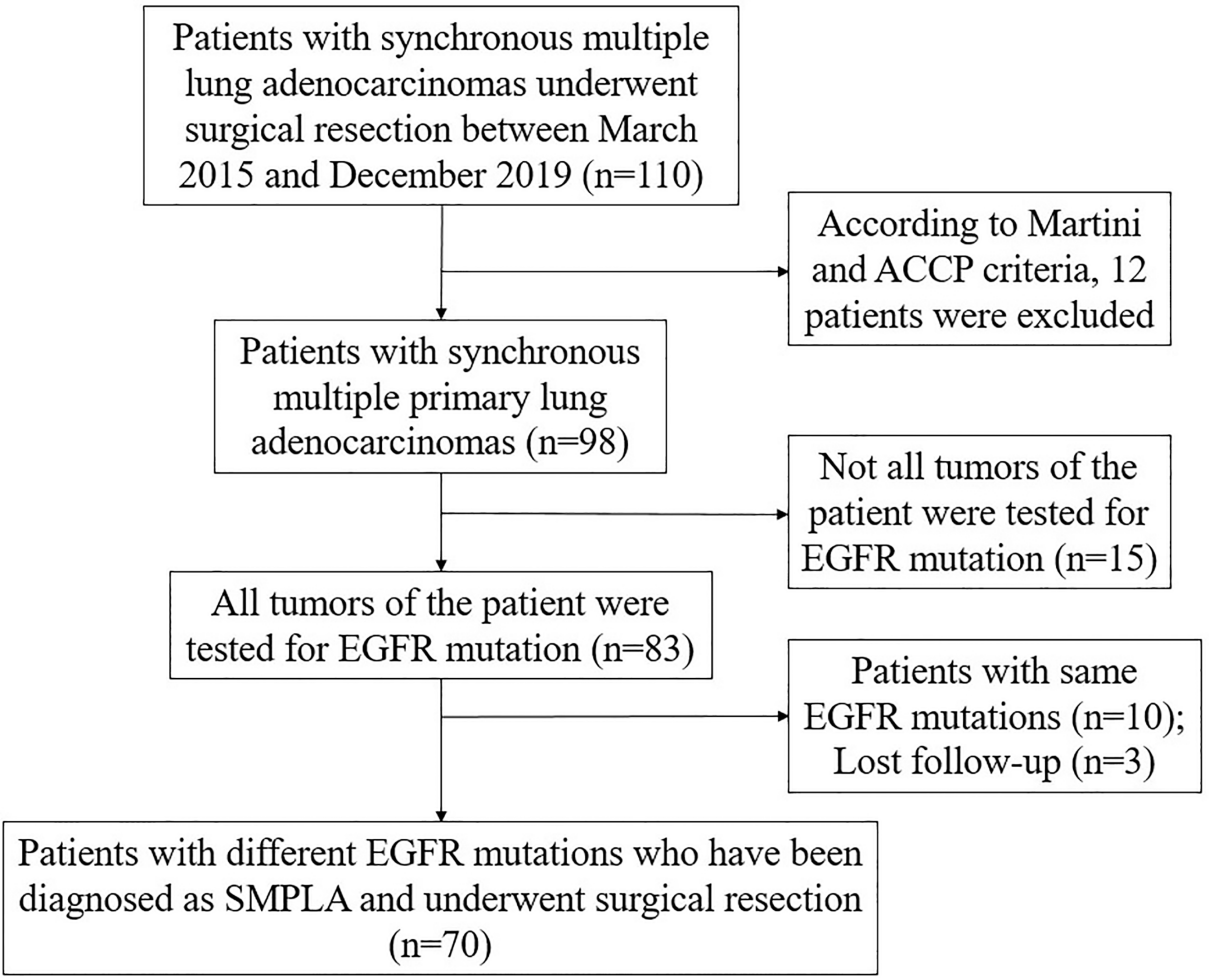
Flowchart of participant selection.

This study retrospectively analyzed the clinical data of patients with SMPLA who underwent simultaneous surgical resection in the Department of Thoracic Surgery at Wuhan Tongji Hospital from March 2015 to December 2019. The criteria for diagnosis of SMPLA in this study are based on the Martini-Melamed criteria (8) and incorporate elements of the new international multidisciplinary lung adenocarcinoma classification (15): (1) major histologic subtypes of tumors are significantly different; (2) major histologic subtypes are similar, but all tumors have lepidic growth component to a certain proportion, or immunohistological features or genetic profiles of tumors are different. The inclusion criteria were as follows: (1) number of lesions ≥2; (2) all lesions of the patient were tested for EGFR and the mutations were different; (3) postoperative pathology of the patient’s lesions were all lung adenocarcinoma; (4) the patient did not have adjuvant therapy before surgery; (5) cardiopulmonary function was acceptable and patients could tolerate surgery; (6) no previous history of tumors; (7) no distant metastases on preoperative examinations. The exclusion criteria were as follows: (1) incomplete patient data information; (2) the postoperative pathology of the lesion is not lung adenocarcinoma. Flowchart of participant selection was shown in **Figure 1**. This study was approved by the institutional review board of Tongji Medical College of Huazhong University of Science and Technology and and consent was given by all patients before their clinical records were used.

### Surgical Approach

All patients underwent combined intravenous and inhalation general anesthesia with double-lumen endotracheal intubation to maintain single-lung ventilation. The surgery was performed using a 3cm small single-port approach: a 3cm incision was made between the 5th ribs in the mid-axillary line of the patient’s surgery side to place a thoracoscope, an elbow laparoscopic suction device, electrocoagulation hooks, and a bipartite clamp was placed to hold the lung lobe if necessary. In bilateral surgery, one side of the surgery is completed and the contralateral surgery is performed in the same way. Systemic lymph node dissection was performed if the dominant lesion was diagnosed as invasive tumor by intraoperative rapid frozen pathology. At the end of the operation, pleural drainage tubes were placed in pleural cavity. The patient received a chest radiograph on the second day after surgery, and pleural drainage tubes could be removed if there was no active bleeding or air leakage. Our specific surgical strategy was: (1) for lesions that were all in the same lobe, we performed direct lobectomy; (2) for lesions that are on the same side but not in the same lobe, we performed anatomical lobectomy or segmentectomy for the dominant lesion ≥2 cm, and sublobar resection for the remaining lesions as much as possible; (3) In patients with multiple lesions in both lungs, we give preference to the side with less lung tissue removed to start the procedure, but if the left side is the dominant lesion and lobectomy is required, the surgery should start on the right side. (4) For all peripheral lesions, intraoperative rapid pathology should be performed as much as possible, and the extent of resection should be determined based on the rapid pathology results and imaging of the lesion, but sublobar resection should be performed as much as possible. (5) For GGO lesions, try to adopt sublobar resection as much as possible. In conclusion, we should take into account the characteristics of the tumor, the patient’s physical condition, and the decisions of the physician to try to develop the best individualized treatment plan for each patient. Specific surgical procedures and strategies for selecting the extent of surgical resection are described in our previous study (16, 17).

### Tissue Samples and EGFR Mutation Analysis

Genomic DNA was extracted from Formalin-fixed paraffin-embedded (FFPE) samples using QIAamp DNA Tissue Kit (Qiagen, Germany). EGFR mutation was detected using commercially available kits from YZY Medical (Wuhan, China) based on amplification refractory mutation system real-time polymerase chain reaction technology. Twenty-nine kinds of EGFR mutation in exon 18-21 were detected in all lesions of these patients.

### Follow-Up

Follow-up was performed by outpatient or telephone follow-up. The follow-up time was calculated from the day after surgery and was followed up until November 2020. In the first year after surgery, chest CT, tumor markers and abdominal ultrasound were reviewed every 3 months; in the second year after surgery, the above indicators were reviewed every 6 months; the above indicators were reviewed annually.

### Statistical Analysis

Measured data were expressed as mean ± standard deviation (SD) and differences between groups were analysed by t-tests. Counted data were expressed as number or percent, and differences were analysed using X^2^ or Fisher’s exact tests. The above data was analyzed using Statistical Product and Service Solutions (version 23; SPSS Inc., Chicago, IL, USA). OS was defined as the time from surgery until death from any cause or last follow-up. Recurrence-free survival (RFS) was defined as the time from surgery until recurrence, death from any cause, or last follow-up. The Kaplan–Meier method was used to analyze OS and RFS by GraphPad Prism software version 7.0. P<0.05 was considered statistically significant.

## Results

### Clinical Characteristics of Patients and Tumors

Clinical characteristics of patients and tumors are shown in **Tables 1**, **2** respectively. Based on the inclusion criteria, 70 patients were ultimately included in this study, of whom 48 were female (68.6%). The mean age of patients was 58.6 ± 8.40 years (range, 41-74 years). A total of 16 patients had a history of smoking or were current smokers, and the rest had no history of smoking. Eleven patients had a family history of cancer, mainly lung cancer (7 patients). Twenty-six patients had preoperative co-morbidities, mainly hypertension and diabetes mellitus. Ten patients had a preoperative mild increase in serum CEA. All patients had a good preoperative cardiopulmonary assessment and could tolerate the procedure. Of these patients, 12 had lesions in both lungs, 11 had three lesions, and 5 had no less than four lesions. Sixty-seven patients had GGO lesions, and 3 patients had only solid nodules. The lesions of 47 patients were located in different lobes. A total of 161 lesions with an average diameter of 20.67 ± 11.7 mm; 84.4% were GGO lesions (pGGO, 42.2%; mGGO, 42.2%) and 80.1% of the lesions were ≤20 mm in diameter. Of all lesions, 71.4% were located in the right lung, with the most lesions in RLL (39.8%), the least in RML (11.2%), and the second least in LLL (11.8%). The postoperative pathology of all lesions was dominated by invasive adenocarcinoma (59.6%), followed by *in situ* adenocarcinoma and microinvasive adenocarcinoma. EGFR mutations were present in 67.1% of the lesions, with L858R (35.4%) and 19Del (20.5%) mutations predominating. Among 70 patients, the highest pathological T stage was mainly pT1 (81.4%) and only seven patient had lymph node metastasis.

**Table 1:** Clinical characteristics of patients.

Variables	Number (%)	Mean value
**Age (years)**		**58.6±8.40**
** ≥60**	**29 (41.4)**	
** <60**	**41 (58.6)**	
**Sex**		
** Male**	**22 (31.4)**	
** Female**	**48 (68.6)**	
**Smoking status**		
** Current and former**	**16 (22.9)**	
** Never**	**54 (77.1)**	
**Family history of tumor**		
** Yes**	**11 (15.7)**	
** No**	**59 (84.3)**	
**Comorbidity**		
** Yes**	**26 (37.1)**	
** No**	**44 (62.9)**	
**Preoperative CEA level**		**3.42±1.33**
** ≥5.0 ng/ml**	**10 (14.3)**	
** <5.0 ng/ml**	**60 (85.7)**	
**Ejection fraction**		**63.23±3.57**
** ≥60**	**58 (82.9)**	
** 55-59**	**12 (17.1)**	
**FEV1 (L)**		**2.74±0.53**
** p-FEV1%**		**97.34±15.91**
** ≥100**	**18 (25.7)**	
** 80-100**	**39 (55.7)**	
** ≤80**	**13 (18.6)**	
**Distribution of tumors**		
** Unilateral**	**58 (82.9)**	
** Bilateral**	**12 (17.1)**	
**Number of tumors**		
** 2**	**54 (77.1)**	
** 3**	**11 (15.7)**	
** ≥4**	**5 (7.2)**	
**Highest pT**		
** T1**	**57 (81.4)**	
** T2**	**13 (18.6)**	
**Highest pN**		
** N0**	**63 (90.0)**	
** N1-2**	**7 (10.0)**	
**Adjuvant therapy**		
** Yes**	**8 (11.4)**	
** No**	**62 (88.6)**	

**Table 2:** Clinical data of tumors.

Variables	Number (%)	Mean value
**Total number of tumors**	**161**	
**Tumor characteristics (mm)**		
** pGGO**	**68 (42.2)**	**12.52±8.06**
** mGGO**	**68 (42.2)**	**19.27±8.21**
** SN**	**25 (15.6)**	**31.29±11.88**
**Size of tumors (mm)**		**20.67±11.7**
** ≤10mm**	**70 (43.5)**	
** <10mm, ≤20mm**	**59 (36.6)**	
** >20mm**	**32 (19.9)**	
**Tumor type pattern per patient**		
** Multiple pGGO**	**11 (15.7)**	
** Multiple mGGO**	**9 (12.9)**	
** pGGO+mGGO**	**30 (42.9)**	
** SN+GGO**	**17 (24.3)**	
** Multiple SN**	**3 (4.2)**	
**Location of tumors**		
** RUL**	**64 (39.8)**	
** RML**	**18 (11.2)**	
** RLL**	**33 (20.4)**	
** LUL**	**27 (16.8)**	
** LLL**	**19 (11.8)**	
**Location of lobe**		
** Same lobe**	**23 (32.9)**	
** Different lobe**	**47 (67.1)**	
**Histology in all tumors**		
** AIS**	**36 (22.4)**	
** MIA**	**29 (18.0)**	
** IAC**	**96 (59.6)**	
**EGFR in all tumors**		
** WT**	**53 (32.9)**	
** Mutation**	**108 (67.1)**	
** L858R**	**57 (35.4)**	
** 19Del**	**33 (20.5)**	
** Double mutations** ^**a**^	**5 (3.1)**	
** other**	**13 (8.1)**	

SN, solid nodule; pGGO, pure ground-glass opacity; mGGO, mixed ground-glass opacity; RUL, right upper lobe; RML, right middle lobe; RLL, right lower lobe; LUL, left upper lobe; LLL, Left lower lobe; AIS, adenocarcinoma in situ; MIA, minimally invasive adenocarcinoma; IAC, invasive adenocarcinoma.

a There are two types of mutations in a tumor.

### Surgical Procedure and Perioperative Results

Twelve and 58 patients respectively underwent bilateral and unilateral surgical resection. The postoperative complications included 3 cases of pulmonary infection, 2 cases of atrial fibrillation, and persistent air leakage for more than 3 days was observed in 4 cases. After treatment, they were all discharged smoothly. No severe perioperative complications or deaths occurred. The average operation time was 205.88 ± 61.94 minutes, the average intraoperative blood loss was 273.83 ± 238.60 ml, the mean postoperative daily drainage of chest tube was 163.52 ± 29.46 ml, the mean postoperative chest tube duration was 6.76 ± 3.43 days, and the average postoperative hospital stay 8.43 ± 3.56 days. Details of surgical procedure are described in **Table 3**.

**Table 3:** Surgical procedure and perioperative results of patients.

Variables	Number
**Surgical procedure**	
**Unilateral**	**58**
**Single lobectomy**	**19**
**Lobectomy-wedge resection**	**13**
**Wedge resection-wedge resection**	**8**
**Segmentectomy-wedge resection**	**5**
**Lobectomy-lobectomy**	**5**
**Lobectomy-segmentectomy**	**4**
**Segmentectomy-segmentectomy**	**2**
**Single segmentectomy**	**2**
**Bilateral**	**12**
**Segmentectomy-wedge resection**	**5**
**Lobectomy-wedge resection**	**4**
**Segmentectomy-segmentectomy**	**1**
**Lobectomy-segmentectomy**	**1**
**Lobectomy+segmentectomy-wedge resection**	**1**
**Perioperative results**	
**Operation time (min)**	**205.88±61.94**
**Intraoperative blood loss (ml)**	**273.83±238.60**
**Postoperative chest tube duration (day)**	**6.76 ± 3.80**
**Daily drainage of chest tube (ml)**	**163.58 ± 28.93**
**Postoperative hospital stay (day)**	**8.43 ± 3.56**

### Detail of EGFR Mutation in 161 Tumors of 70 Patients

EGFR detection of all lesions revealed that 108 lesions had mutations, mainly L858R and 19DEL, and their mutation rates were 35.4% and 20.5%, respectively. Among the different radiologic features, the mutation rate of mGGO was significantly higher than that of pGGO and SN (P<0.001); among the different histology features, the mutation rate of invasive adenocarcinoma was significantly higher than that of other histology subtypes (P<0.001); among the different size, the mutation rate for lesions >20 mm was significantly higher than that of lesions ≤20 mm (P<0.001). However, the mutation rate of specific types were not significantly different among radiologic features, pathology types, or sizes (P>0.05). The results of EGFR mutation are presented in **Table 4**.

**Table 4:** Distribution of EGFR mutations in 161 tumors of 70 patients.

Variables	Total	WT	EGFR+	P-value	L858R	19Del	Other^a^	P-value
**Different radiology**								
**pGGO**	**68**	**34 (50.0)**	**34 (50.0)**		**15 (44.1)**	**14 (41.2)**	**5 (14.7)**	
**mGGO**	**68**	**13 (19.1)**	**55 (80.9)**		**33 (60.0)**	**14 (25.5)**	**8 (14.5)**	
**SN**	**25**	**6 (24.0)**	**19 (76.0)**	**0.000**	**9 (47.4)**	**5 (26.3)**	**5 (26.3)**	**0.386**
**Different histology**								
**AIS**	**36**	**22 (61.1)**	**14 (38.9)**		**7 (50.0)**	**5 (35.7)**	**2 (14.3)**	
**MIA**	**29**	**13 (44.8)**	**16 (55.2)**		**8 (50)**	**5 (31.3)**	**3 (18.7)**	
**IAC**	**96**	**18 (18.7)**	**78 (81.3)**	**0.000**	**42 (53.8)**	**23 (29.5)**	**13 (16.7)**	**0.959**
**Different size**								
**≤10mm**	**70**	**34 (48.6)**	**36 (51.4)**		**17 (47.2)**	**12 (33.3)**	**7 (19.5)**	
**<10mm, ≤20mm**	**59**	**16 (27.1)**	**43 (72.9)**		**24 (55.8)**	**14 (32.6)**	**5 (11.6)**	
**>20mm**	**32**	**3 (9.4)**	**29 (90.6)**	**0.000**	**16 (55.2)**	**7 (24.1)**	**6 (20.7)**	**0.749**

SN, solid nodule; pGGO, pure ground-glass opacity; mGGO, mixed ground-glass opacity; AIS, adenocarcinoma in situ; MIA, minimally invasive adenocarcinoma; IAC, invasive adenocarcinoma; ^a^Refers to other rare mutations including L861Q, G719X, 20Ins and T790M.

### Postoperative Treatment and Follow-Up of Patients

**Figure 2:**
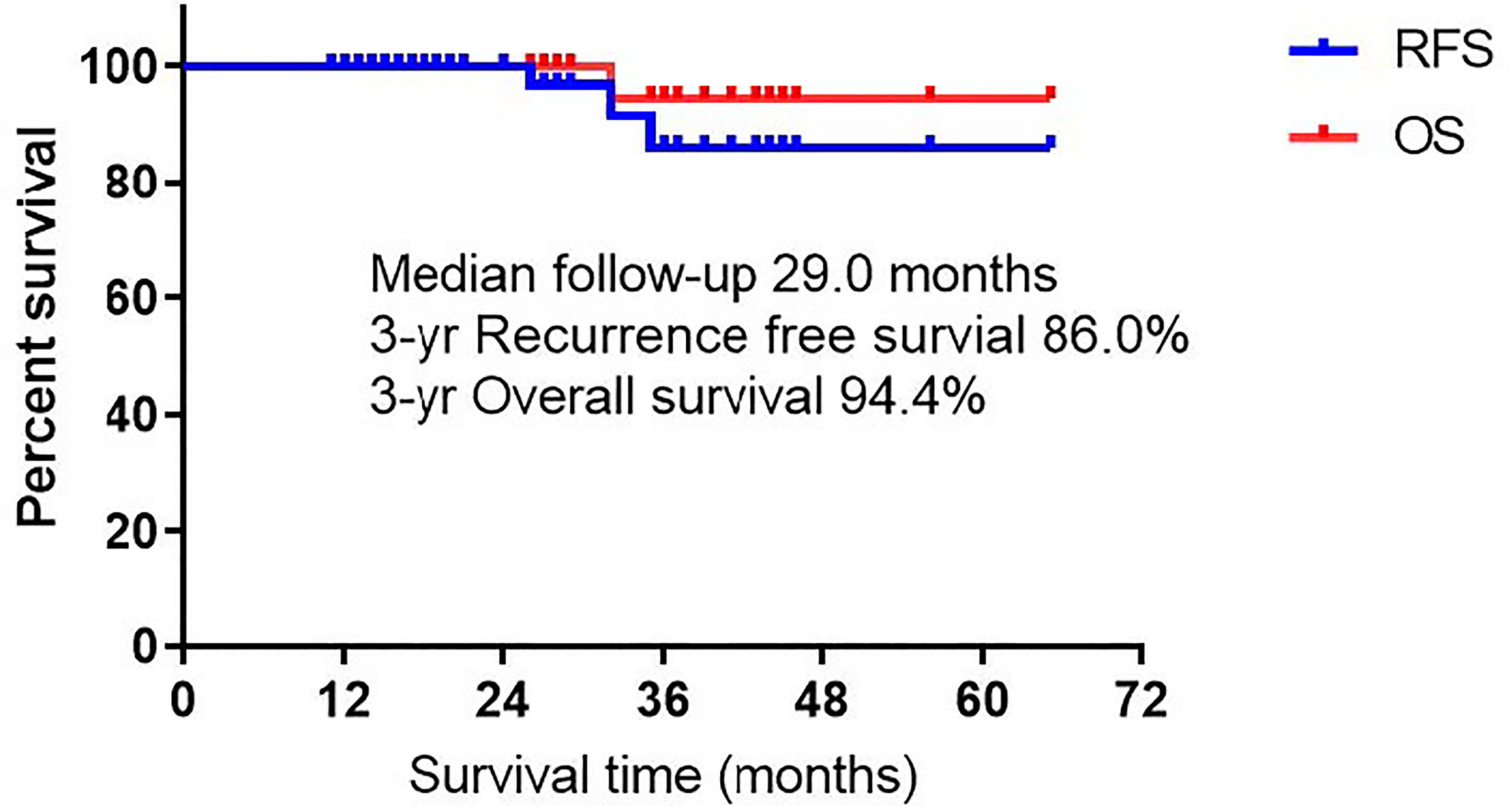
Recurrence free survival and overall survival of all patients.

Eight patients received adjuvant therapy after surgery, of which seven patients received targeted therapy due to the presence of lymph node metastases, and one patient received chemotherapy because the lesions invaded the pleura and were larger than 4 cm. As of November 30, 2020, the average follow-up time was 30.6 ± 13.5 months. Except for three patients with recurrent metastases, one of whom died due to extensive postoperative pleural metastases, the rest of the patients did not develop new lesions or metastases, and all of them are alive. The 3-year OS and RFS in all patients were 94.4% and 86.0%, respectively (**Figure 2**).

## Discussion

Lung cancer has been a leading cause of cancer-related death worldwide for decades, with adenocarcinoma representing the most prevalent subtype (18). In recent years, with the popularity of low-dose spiral CT screening, more and more SMPLC are being diagnosed. At the same time, research on the diagnosis and treatment of multiple primary lung cancers has increased significantly. It is difficult to distinguish multiple primary lung cancers from intrapulmonary metastases, but the treatment options and prognosis of multiple primary lung cancers are completely different compared with intrapulmonary metastases, so the evaluation of diagnosis, treatment, and prognosis of multiple primary lung cancers is particularly important. Unlike previous studies, in this study, we retrospectively examined the clinical characteristics, surgical treatment, and long-term prognosis of all patients with postoperative lesions diagnosed as multiple primary lung adenocarcinoma by EGFR testing. We found a high heterogeneity of EGFR driver genes between tumors in patients with multiple primary lung adenocarcinoma, suggesting the importance of EGFR testing in the diagnosis of such patients.

In the present study, we classified lesions by their radiology, pathology type, and diameter size of the lesions, and then compared EGFR mutations in lesions in different categories. We found that the mutation rate of mixed GGO is significantly higher than that of pure GGO and solid nodules (SN); the mutation rate of invasive adenocarcinoma is significantly higher than that of other histology subtypes (AIS as well as MIA). These results are in general agreement with the findings of Liu et al. (19). The result also concurred with the hypothesis for the progression of lung adenocarcinoma that EGFR-mutated AAH follows a linear progression schema, whereby AAH progresses to AIS and is followed by MIA (20, 21). Sun and colleagues (22) found that diameter size of GGO lesion correlated with EGFR mutation rates, with lesions ≥20 mm in diameter being more likely to be mutated than lesions <20 mm in diameter, which is similar to the results of our study.

For multiple primary lung adenocarcinoma, we should routinely test for EGFR mutations in all lesions. Although multiple lung cancers with predominantly multiple GGO lesions or containing GGO lesions should be considered more often as multiple primary lung cancers, one study found the presence of similar somatic mutations by exon sequencing in multiple GGO lesions in two patients with multiple lung adenocarcinomas, including two pure GGO lesions in one patient (23). The result suggests that intrapulmonary metastases can occur in patients with multiple GGO lesions. In the current study, all but three patients had solid lesions, and the rest contained at least one GGO lesion, yet postoperative pathology showed lymph node metastases in seven patients, all of whom should be considered to have multiple intrapulmonary metastases according to previous Martini-Melamed criterion (8). Ye and colleagues (24) reported a case of a patient with multiple primary lung adenocarcinoma in whom two tumors, one with EGFR mutation and one with KRAS mutation, were identified by genetic testing, and the lesion with the KRAS mutation was resected and followed by gefitinib-targeted therapy, after which the remaining lesion disappeared. Therefore, we believe that EGFR mutation can be a good supplement to histological, imaging and morphological evidence of tumor, so as to better distinguish multiple primary lesions from metastatic lesions and provide patients with a more accurate staging. In this study, analysis of EGFR testing results for all lesions revealed the presence of EGFR mutations in 108 lesions (67.1%), including 35.4% and 20.5% for L858R and 19DEL, respectively. This may be related to the fact that the patients in this study were non-smokers (77.1%) and the majority of female patients (68.6%).

Surgery remains the most effective treatment option for multiple primary lung cancers, but the specific surgical method is still controversial. Several studies (7, 25–27) have shown that for multiple primary lung adenocarcinoma, lobectomy should be performed as far as possible for the primary lesion, while sublobar resection (segmentectomy or wedge resection) can be performed flexibly for the secondary lesion, especially for patients with multiple bilateral lung lesions, which ensures adequate distance between the tumor margins and maximizes preservation of more lung function. Nakata and colleagues reported (25) that 26 patients with SMPLA, only 5 patients underwent lobectomy alone, and the 3-year OS and DFS were 92.9% and 77.9%, respectively. Ishikawa and colleagues also found (7) that 93 patients with SMPLA, sublobar resection was used during surgery in 58% of patients, and the 3-year OS and RFS were 93.6% and 87% respectively. In the current study, since most of lesions were distributed in different lobes, we tried to adopt a combined sublobar resection approach during surgery, and the OS and RFS at 3 years reached 94.4% and 86%, respectively, which was comparable to the results of the above study.

There are several limitations to our study. First, it is a retrospective study and selection bias cannot be avoided. Second, it is a single-center study with a small sample size, which needs to be further confirmed by multicenter study with larger sample size. However, our study is currently the largest cohort of SMPLA with different EGFR mutations. Third, we did not test other tumor’s driver genes, such as KRAS, ALK, ROS1, and BRAF. A whole genome sequencing would be more accurate to identify the source of multiple tumors. Finally, the follow-up time is not long enough to appropriately assess long-term survival. In future studies, we will provide longer-term follow-up data.

In summary, a high discordance of EGFR mutations were identified between tumors in patients with SMPLA, so the detection of EGFR mutation may be used routinely to prevents unnecessary adjuvant treatment for patients with histologically similar synchronous primary lung cancers. Synchronous multiple lung adenocarcinomas with predominantly multiple GGO should be considered as SMPLA, and surgery may be aggressively performed for these patients due to a good prognosis.

## Data Availability Statement

The original contributions presented in the study are included in the article/supplementary material. Further inquiries can be directed to the corresponding author.

## Ethics Statement

The studies involving human participants were reviewed and approved by the institutional review board of Tongji Medical College of Huazhong University of Science and Technology. The patients/participants provided their written informed consent to participate in this study.

## Author Contributions

RQ, YC, and XF contributed to the design of the study and the performing of the procedure. RQ, FY, and DT acquired and analyzed the data. RQ drafted the manuscript. RQ, FY, DT, and XF revised and edited the manuscript. All authors contributed to the article and approved the submitted version.

## Funding

This work was supported by the Tongji Hospital Clinical Research Flagship Program (No. 2019CR107).

## Conflict of Interest

The authors declare that the research was conducted in the absence of any commercial or financial relationships that could be construed as a potential conflict of interest.

## Publisher’s Note

All claims expressed in this article are solely those of the authors and do not necessarily represent those of their affiliated organizations, or those of the publisher, the editors and the reviewers. Any product that may be evaluated in this article, or claim that may be made by its manufacturer, is not guaranteed or endorsed by the publisher.
